# Transfer learning for cross-context prediction of protein expression from 5’UTR sequence

**DOI:** 10.1093/nar/gkae491

**Published:** 2024-06-12

**Authors:** Pierre-Aurélien Gilliot, Thomas E Gorochowski

**Affiliations:** School of Biological Sciences, University of Bristol, 24 Tyndall Avenue, Bristol BS8 1TQ, UK; School of Biological Sciences, University of Bristol, 24 Tyndall Avenue, Bristol BS8 1TQ, UK; BrisEngBio, School of Chemistry, University of Bristol, Cantock’s Close, Bristol BS8 1TS, UK

## Abstract

Model-guided DNA sequence design can accelerate the reprogramming of living cells. It allows us to engineer more complex biological systems by removing the need to physically assemble and test each potential design. While mechanistic models of gene expression have seen some success in supporting this goal, data-centric, deep learning-based approaches often provide more accurate predictions. This accuracy, however, comes at a cost — a lack of generalization across genetic and experimental contexts that has limited their wider use outside the context in which they were trained. Here, we address this issue by demonstrating how a simple transfer learning procedure can effectively tune a pre-trained deep learning model to predict protein translation rate from 5’ untranslated region (5’UTR) sequence for diverse contexts in *Escherichia coli* using a small number of new measurements. This allows for important model features learnt from expensive massively parallel reporter assays to be easily transferred to new settings. By releasing our trained deep learning model and complementary calibration procedure, this study acts as a starting point for continually refined model-based sequence design that builds on previous knowledge and future experimental efforts.

## Graphical Abstract

**Graphical Abstract:**
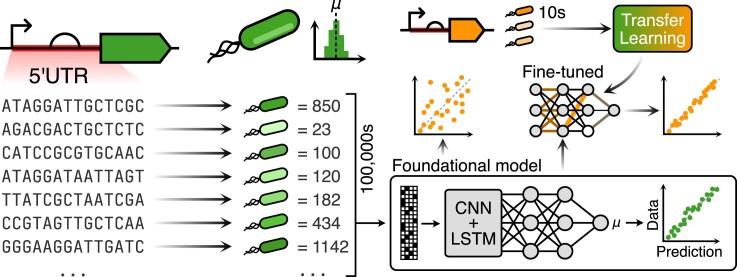

## Introduction

When engineers build a device or system they typically assume that the parts available to them perform in a reliable and well-defined way. This robustness and modularity allows for model-based design of large and complex systems, as individual parts can be characterized in isolation and the function of their composition accurately predicted by mathematical models. Synthetic biology has attempted to adopt this approach, with libraries of genetic parts being created, curated and reused across the field (1,2). However, numerous experiments have shown that biological parts often do not behave consistently when used in different ways and often lack the robust modularity that underpins model-based design approaches (3). Multiple factors have been identified as contributing to these contextual effects. These include: genetic/compositional (4–6), functional (7–9), host-related (10–12), environmental (13–16) and experimental aspects (17). This difficult to predict variability in part function causes genetic constructs to often fail when first built and leads to time-consuming redesign and tweaking before a working system is found (18).

The 5’ untranslated region (5’UTR) of a transcript plays an important role in controlling the translation initiation and mRNA stability (19), and has been found to be particularly prone to variability in nearby sequences (i.e., sequence context), especially in prokaryotes. This makes the 5’UTR a versatile platform for controlling gene expression by altering: (i) sequences upstream of the ribosome binding site (RBS) (5), (ii) sequences within the RBS itself (20–22) and (iii) synonymously re-coding the N-terminal codons of the protein coding sequence downstream of the RBS (23). Interactions between all of these elements and the formation of secondary structures can alter protein expression rate through various mechanisms, including ribosome binding occlusion and pausing (21,24–26).

While these contextual effects offer a rich foundation for evolution to shape gene expression, they also make predictive model-guided design difficult because genetic parts are typically characterized in a limited (often only one) context. Two strategies are therefore commonly used to enable effective genetic circuit design. The first introduces design features that improve the robustness of part function (27). For example, adding insulating sequences (2,28,29) and parts (e.g., self cleaving ribozymes) (1,30,31) to expression cassettes has proven valuable in reducing compositional effects (2,31,32), and at the functional level, feedback control can be implemented to reduce interference between modules (33–39). However, the limited number of insulating elements and the additional metabolic cost of implementing feedback compensating mechanisms or tunable systems (40,41) means these approaches are not always viable.

A second approach is to not avoid contextual effects, but embrace them by attempting to understand and/or exploit them (42,43). One way to do this is by using emerging high-throughput assembly and characterization approaches (44–47) to build large libraries of parts that are then screened to find variants specifically tailored for the task and context at hand. While appealing, difficulties in assembling sufficiently diverse libraries (48) and the prohibitive cost of assaying their function, means that this methodology is challenging and rarely used.

In contrast, to better understand contextual effects, mechanistic models like the RBS Calculator (22), OSTIR (49) and RBSeval (50) have been developed to predict the effect that local sequence composition in the 5’UTR and protein coding region has on translation initiation rate. However, our limited understanding of the underlying biophysical processes often hampers the expressivity of mechanistic models, preventing them from providing accurate predictions in contexts where new biophysical phenomena play a role (51,52). To overcome these limitations, large genotype-to-phenotype datasets (44,51,53) have also been used to employ deep-learning techniques for prediction of part function from sequence alone (54–56). However, despite their performance, these models also suffer from poor generalization as they only see data from the biological context used for training. While it would be possible to acquire data across larger numbers of contexts using high-throughput methodologies like Flow-seq (44,51,53) and uASPIRE (54) to improve model generalization, such experiments are extremely costly, laborious and typically out of reach for most labs (57).

A solution to this problem could be to measure a smaller subset of designs that fit within existing experimental workflows (e.g., in a 96-well plate) to provide a limited set of supplementary training data from the new context. Training a deep learning model from scratch on this small dataset would likely lead to poor performance (58). However, a successful technique in computer vision known as transfer learning (59) where models are reused across tasks could be used to remove the need to train from scratch. Models like ConvNets (60) are typically pre-trained on large datasets such as ImageNet (61) and then fine-tuned for specific tasks where data is scarce. Compared to training using a random initialization of the neural network, fine-tuning of a pre-trained model offers superior performance while requiring significantly less data. Transfer learning applied to deep learning models of sequence-to-function relationships could have the potential to create many tailored models for each context that can be used during the design phase.

**Figure 1.**
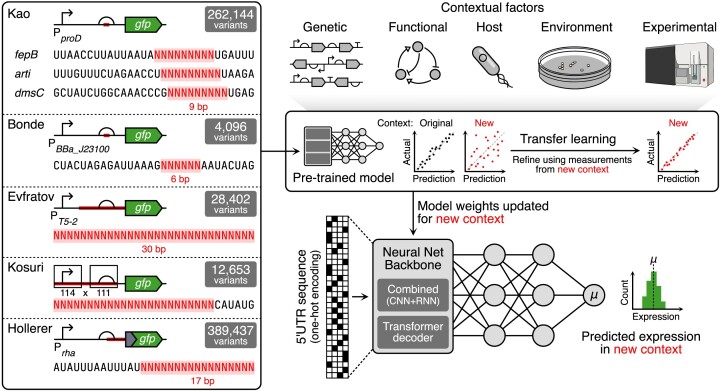
Transfer learning pipeline to adapt pre-trained models for new contexts. Left box contains the design of the datasets considered for model pre-training and testing. Randomized sections of the 5’UTR are shown in red. On the right-hand side, top elements indicate the various contextual factors that can affect the function of a genetic part. Middle box shows the workflow we use, starting with a pre-trained deep neural network and then fine-tuning this model via a transfer learning approach with a small number of samples from a new context in which predictions need to be made. Lower region shows the general structure of the different neural networks considered (either CNN-LSTM or the frozen transformer decoder backbone from the RNA foundational model), which predict mean protein expression from a 5’UTR sequence.

In this work, we use transfer learning to adjust the weights of a pre-trained deep learning model able to predict protein expression from 5’UTR sequence in *Escherichia coli* for a range of new contexts (Figure 1). We focus predominantly on varying contextual effects related to sequence composition. However, other experimental contextual effects also vary between the datasets we study with different strains of *E. coli*, a variety of cell sorting machines and approaches for library characterization, and a variety of culturing conditions being used. We demonstrate the effectiveness of a combined neural network architecture that includes both convolutional and long short-term memory units (CNN-LSTM) to model the effects of base pair mutations in 5’UTR sequences and show that the features learned during training can be adapted to different sequence and experimental contexts using a simple transfer-learning procedure. Using this approach, we quantify the amount of data needed to successfully transfer models across a range of different contexts to identify the appropriate data calibration procedure. Our pre-trained model and simple fine-tuning pipeline are a valuable tool to support model-based 5’UTR engineering, and demonstrate that transfer learning may provide a means for applying data-centric models in biological design.

## Materials and methods

### Dataset preparation

We inferred the log-normal distribution parameters for the fluorescence distributions of each sequence in the Kuo (20), Kosuri (5) and Evfratov (62) datasets using the FORECAST package (57,63). The maximum fluorescence of the last sorting bin was set to 10^5^. The precision associated with each dataset was assessed using the width of the median 99.7% confidence interval obtained during the inference procedure. The median was used instead of the mean to avoid the outliers displaying abnormally large confidence intervals. Except for the Evfratov dataset, only constructs sorted into multiple bins and with an invertible hessian at their maximum likelihood estimate were kept. We did not perform inference on the Bonde dataset as we could not access the raw Flow-seq sequencing data. Instead, we relied on the green fluorescent protein (GFP) expression estimates provided in the original paper (64). Since the Hollerer dataset was curated using a methodology different from Flow-seq (i.e. uASPIRE) (54), we also incorporated their GFP expression estimates in our analysis.

The Bonde, Kuo and Hollerer datasets were divided into training, validation, and test sets using a random split approach. The splitting ratios for these sets were 80% for training, 10% for validation, and 10% for testing. Stratified sampling was employed to ensure that the distribution of fluorescence levels was similar for the original dataset and each of the subsets. This was achieved by binning of the data based on fluorescence and then sampling from those bins where the probability of drawing a sample from a bin is proportional to the number of elements within it. For the Kosuri dataset, we filtered out RBSs used to train the RBS Calculator (22) to avoid unfair comparisons. We discarded promoters with less than 60 RBSs after the filtering steps. For each of the 63 promoters that remained, we assembled the corresponding test set by first selecting 30 sequences, with balanced log-fluorescence means, and split the remaining sequences in the validation set (always 20 sequences) and the training set (ranging from 12 to 61 sequences according to the promoter). The spread of RBS strengths for each dataset was quantified by the 1st to 99th quantile range. The margin of tolerance was then calculated as a quarter of this range, allowing constructs to be classified into one of four possible categories uniformly separated in log-fluorescence space: i.e., weak, medium weak, medium strong and strong. The width of each category was 1.12 for the *fepB* dataset, 1.06 for the *arti* dataset, 1.28 for the *dmsC* dataset, 0.22 for the Bonde dataset and 1.6 for the Kosuri dataset.

### Activity cliff labeling

Test sequences from the *fepB* dataset were split into two groups based on their log-fluorescence mean. 5’UTR sequences below a value of 3 were classified as weak and sequences above a value of 6 were classified as strong. We then processed the weak and strong RBSs and labeled activity cliffs as pairs of sequences differing by a single nucleotide. This resulted in 388 sequences for the *fepB* test set. To obtain the RBS calculator v2.1 predictions for each activity cliff, we ran each 5’UTR sequence followed by the next 60 nt of the coding sequence through the web interface in batch mode. From the data returned, we selected the translation initiation rate (TIR) at position 30, which corresponded to the start codon of the reporter gene. These TIR values were then used as the prediction.

### Model architecture and hyperparameter optimization

Each model used as input the one-hot encoded 5’UTR sequence, followed by a particular backbone (LSTM, CNN or CNN-LSTM), resulting in an output matrix that was flattened and then followed by a dense multi-layer perceptron (MLP) to predict the target output value. For the CNN-LSTM model, the output of the CNN layer was a matrix of size [features, step], which was used as input to the LSTM layer. Models predicting the mean log-fluorescence for each 5’UTR were trained by minimizing the mean squared loss. We optimized the batch size, learning rate and architecture parameters for each candidate model (CNN or CNN-LSTM) to minimize the loss on the validation dataset. Learning rate was updated according to the ReduceLROnPlateau scheduler (65) with parameters: mode = ‘min’, factor = 0.5, patience = 3, threshold = 0.01, min lr = 10^−5^. Models were trained for a maximum of 20 epochs and the loss on the validation dataset was monitored in order to use early stopping with a patience of 5 epochs to prevent over-fitting. Hyperparameters for the CNN and CNN-LSTM models trained on the *fepB* dataset were optimized using the Tree-structure Parzen estimator implemented in Optuna (66). We used a budget of 200 trials to optimize each architecture. Unpromising trials were pruned based on the intermediate value of the loss on the validation dataset. Parameter search space and optimal values for each architecture are noted in Supplementary Table S1.

### Scaling experiments

To assess the impact of the training set size on the model performance, we trained the CNN-LSTM model with the optimal hyperparameters on a subset of the training and validation data. The test dataset size did not vary. The total number of fine-tuning examples started at 10 and were then log-spaced to reach the maximum size of each dataset, with 80% of the examples allocated for model training and the remaining 20% for model validation. Experiments were conducted five times for each dataset with different seeds (3, 7, 13, 15, 16) to allow for various dataset splits and different initializations of the neural network parameters.

### Predicting standard deviation of log-normal distributions

The same CNN-LSTM backbone was used to model other distribution parameters for each sequence in the *fepB* dataset with the only difference being that the output layer included an additional neuron to capture the standard deviation σ of the log-normal fluorescence distribution. Following previous work (67), we used the Kullback–Leibler divergence (68) between the predicted and ground truth distribution as the loss function to train the model.

### Transfer learning approaches

Two transfer learning procedures were used to fine-tune each pre-trained model for new contexts. The predominant one consisted of using each pre-trained model and then further training it on the new dataset using the same mean squared loss, however, with a lower learning rate. All neural networks parameters could be updated. We used the ADAM optimizer with a learning rate of 3 × 10^−5^ and a batch size of 32 for a maximum of 600 epochs. The learning rate was updated according to the ReduceLROnPlateau scheduler with parameters: mode = ‘min’, factor = 0.5, patience = 3, threshold = 0.01, min lr = 10^−5^. We monitored the loss on the validation dataset and used early stopping with a patience of 80 epochs to prevent over-fitting. For the second transfer learning approach, we froze all parameters of the backbone CNN-LSTM model up to the final MLP. The perceptron for this model consisted of one intermediate layer with 128 nodes and a single output node. Only parameters in the MLP were updated during the fine-tuning protocol, using the same approach as described above.

### Evaluation of transfer learning for the Kosuri dataset

The Kosuri test set is small (30 samples for each promoter subset), which prevented accurate estimation of performance metrics. We therefore used the following approach to account for and quantify the measurement uncertainty. The standard error for the accuracy metric was estimated via 500 bootstrap samples of the test data using the quantile method. To detect a statistical difference before and after fine-tuning, we conducted a paired permutation test on each promoter dataset. The null hypothesis was that fine-tuning does not improve protein expression prediction from the sequence. The permutation test function from the MLXTEND package (69) was used with 10 000 permutations to allow for detection of *P*-values as small as 10^−4^.

### RNA foundational model

We used the RNA foundational model (RNA-FM) developed by Chen *et al.* (70), which is based on the BERT bidirectional transformer architecture (71) and trained on RNA-central (72) using a masked language model and self-supervised learning scheme (71). We used this model to generate embeddings of shape (*L*, 640) for each 5’UTR sequence of length *L*. These embeddings were then used as inputs for a downstream architecture comprising 6 residual blocks of 1D convolutional layers followed by a flattening layer and a regression head to predict the mean log-fluorescence. This architecture was trained and fine-tuned on different datasets according to the same protocol described above.

### Alternative non-deep learning models

The RBS Calculator v2.1 web server was used to compute the TIR for each activity cliff in the *fepB* test dataset. However, using the web server to compute the TIR is not amenable to larger datasets (typically >20 000 sequences for the *arti* and *dmsC* contexts). Therefore, we used RBS Calculator v1 that still allows for programmable access to the model and OSTIR (49), an open-source implementation of the RBS Calculator v1. Both of these models were used to compute predictions for the *arti* and *dmsC* test sets. We also considered alternative thermodynamic-based predictors, including the RBSeval model (50), the anti-Shine Dalgarno:Shine Dalgarno (aSD:SD) base pairing free energy and the local mRNA folding free energy, which were both calculated for each 5’UTR in the Kuo dataset.

### Computational tools

All analysis scripts and the deep learning pipelines were written in Python version 3.9 using numpy version 1.19.15 (73) for matrix algebra, scipy version 1.4.1 (74) for statistical functions, and Pytorch version 1.12 (65) for building and training the deep learning models.

## Results

### Model development

We began by developing a deep learning model to predict protein expression rate from 5’UTR sequence. Deep learning models are ideally suited to modelling sequence-to-function relationships due to their ability to learn complex nonlinear mappings to any level of precision. Given their excellent performance in genomic studies (75), we investigated the ability of Convolutional Neural Networks (CNNs) and combined Convolutional-Long Short Term Memory (CNN-LSTM) neural networks to accurately learn the mean gene expression level (log-fluorescence) from 5’UTR sequence alone. Both models were initially trained on a dataset generated by Kuo *et al.* (20), where a fixed 5’UTR sequence contains a 9-nt variable Shine Dalgarno (SD) region (Figure 1). We specifically used the *fepB* subset when developing our models. Because our CNN-LSTM model learns directly from data, it is essential that the quality (i.e., accuracy) of the data used during training and validation is high. We have previously shown that using simple statistics to analyse raw Flow-seq data results in poor quality data that is often biased (57). To avoid this, we used a maximum likelihood-based inference method implemented by the software package FORECAST to improve the quality of the datasets used. Pre-processing the raw Flow-seq data using FORECAST resulted in the mean (μ) and standard deviation (σ) of the underlying log-normal fluorescence distribution (76) being calculated for each 5’UTR sequence.

**Figure 2.**
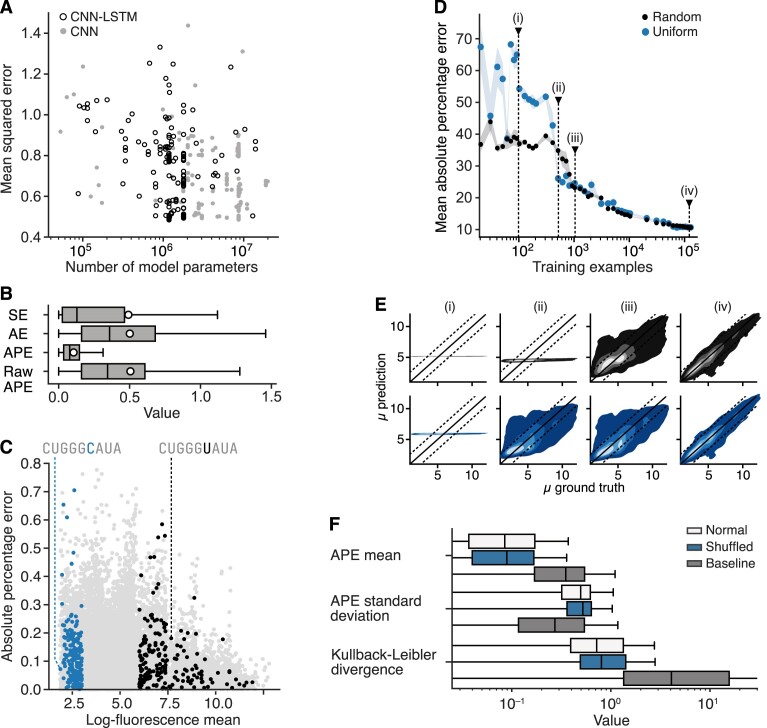
A deep learning model accurately predicts protein expression from 5’UTR sequence. (**A**) Number of parameters and best mean squared error (evaluated on the validation set) of all models trained during the hyperparameter optimization phase using the *fepB* dataset. (**B**) Distribution of the test set performance metrics (SE: squared error, AE: absolute error, APE: absolute percentage error of the log-fluorescence data, raw APE: absolute percentage error of the raw fluorescence data) for the CNN-LSTM model trained to predict the mean log-fluorescence from 5’UTR sequence. (**C**) Distribution of prediction errors for the test dataset. Activity cliffs (pairs of sequences with vastly differing expression levels, but with sequences that have only a single base change) are colored in blue and black. Each sequence in blue has a corresponding sequence in black. Two representative sequences are highlighted with the specific base change highlighted. (**D**) Performance of the best CNN-LSTM model predicting the mean log-fluorescence with varying sizes and compositions of training data. Random (black) corresponds to a training set randomly chosen, while uniform (blue) corresponds to a training set that contains sequences that display a mean log-fluorescence that uniformly covers the range of values present in the entire dataset. Shaded areas denote the standard error of the mean absolute error computed across five models trained with different random seeds to illustrate the variability in performance due to both library acquisition and model training. Four key point are highlighted (i)–(iv). (**E**) Kernel density plots of the test set predictions from a model trained using a random (top) or uniform (bottom) training set containing from left to right, 100, 500, 10^3^ and 10^5^ sequences relating to points (i)–(iv) in panel D. (**F**) Performance evaluation of models trained to simultaneously predict the log-fluorescence mean and standard deviation.

Rather than defining a fixed architecture for our models, we used neural network hyperparameter optimization to find suitable designs that maximised the accuracy of predictions (Materials and methods). This revealed that both types of model could reach a similar level of performance, with a mean squared error (MSE) = 0.49 ±0.01 for the *fepB* dataset. To avoid over-fitting, we employed early stopping during model training, which can be observed on the training/validation learning curves (Supplementary Figure S1). Interestingly, the CNN-LSTM model achieved this performance with eight times fewer parameters than the CNN model, having 1.1 million versus 8.7 million parameters, respectively (Figure 2A). The efficiency of the CNN-LSTM model is likely due to how the convolutional and recurrent layers capture complementary aspects of the gene expression process. CNNs excel at extracting motifs/patterns in sequences that are known to play an important role (e.g., facilitating base pairing between the mRNA and the 3’ end of the 16S ribosomal RNA). In contrast, the LSTM module is able to efficiently capture the ordering and distance between such motifs, which again is known to be critical for controlling translation initiation rate (21). This allows the CNN-LSTM model to exploit patterns in both of these features more efficiently than the CNN can alone. Curiously, the perceptron block of the CNN-LSTM model selected after hyperparameter optimization contained only a single intermediate layer. This suggests a relatively simple relationship between the features extracted by the backbone (CNN and LSTM layers) and the output mean log-fluorescence value.

To assess the model’s performance, it is important to consider the wide range of mean log-fluorescence values observed in the *fepB* dataset (the 1%/99% percentile interval spans (0.9, 11.7)). Using only the MSE metric may not fully capture the magnitude of the error (i.e., small errors for weak 5’UTRs may be as significant as large errors for strong 5’UTRs). We therefore also employed other metrics such as the Mean Absolute Percentage Error (MAPE) to gain better insights into the model’s performance. The best performing CNN-LSTM architecture was found to achieve a MAPE = 10.9% ± 0.1% on the test set (Figure 2B), which is similar to the minimum achievable 9.7% for this dataset. The minimum achievable MAPE was estimated by computing the median width of the 99.7% confidence interval for the MAPE of the mean log-fluorescence, when accounting for the uncertainty occurring during the Flow-seq measurement of the mean log-fluorescence, which was modelled using FORECAST (Supplementary Figure S2). Without the log-transformation of the data, the model error corresponds to a MAPE of 54% ± 2% for the raw fluorescence values (Figure 2B).

Modifying the 5’UTR is a popular approach for controlling gene expression in prokaryotic cells (77,78) because small changes to the sequence can dramatically alter protein expression (20,22,79). Not only is the CNN-LSTM model able to accurately predict those changes on average across the test sequences, it is also able to distinguish between strong and weak 5’UTRs that differ by only a single nucleotide. Indeed, the model had a MAPE of 12% and 11% for the weak and strong expression test sets, respectively (Figure 2C). With the model performing well on the test set, the ability to resolve activity cliffs (pairs of 5’UTR with large differences in protein expression, but where the sequences only differ by a single nucleotide), suggests that our CNN-LSTM model does not overfit to the training set, but is instead able to generalize broadly across the 5’UTR sequence landscape within the *fepB* context. Such nucleotide level sensitivity is difficult to obtain with non-deep learning models, and we typically observed highly similar predictions for the activity-cliff sequences when using biophysical models. Specifically, for the RBS Calculator v2.1 (22), we found that the strong-weak ordering was incorrect in 14% of the pairs. We also observed a high degree of similarity in predictions, as shown by the ratio of high-to-low initiation rates that never exceeded 5 for 74% of the pairs (Supplementary Figure S3).

We also assessed the ability for our model to capture higher order statistical features like the standard deviation. However, as demonstrated previously (54,57), we were unable to generate accurate predictions (Supplementary Note 1). For this reason, we focused our efforts on the mean log-fluorescence, which is typically the most important feature for biological engineers wanting to use these sequences.

### Data volumes required for model training

As high-throughput assays are expensive and time-consuming, we wanted to better understand the minimal number of samples required to train models across a range of contexts. To do this, we performed a data-efficiency analysis of the CNN-LSTM model where we trained models from scratch on sub-sets of the *fepB* training and validation datasets. As expected, we found a consistent improvement in performance as the number of training examples increased, with the MAPE falling from $70\%$ to $10.5\%$ (Figure 2D). Interestingly, a sharp increase in performance was observed at around 500–1000 training examples, indicating a learning phase transition. Initially, the CNN-LSTM output is merely a constant function (the mean training data fluorescence), before the transition is made to the output predicting differing levels of fluorescence across the library (Figure 2E). Understanding how this transition point occurs is crucial to minimize learning requirements. To better understand what this phase transition depended on, we related the location of this transition to the information content of the training dataset. We compared two methods of selecting sequences for this dataset. First, we used random selection, which enriched the dataset with constructs displaying weaker fluorescence due to majority of sequences falling in this region. Second, we selected sequences that displayed a balanced spectrum of fluorescence profiles (i.e. covering weak to strong fluorescence). We found that using a random selection of sequences for training delayed learning and pushed the transition point to around 1000 examples, approximately 500 examples later than when a balanced test dataset is used (Figure 2D).

### Transfer learning between different sequence contexts

The previous sections have shown that a CNN-LSTM based deep learning model can accurately predict how mutations within the RBS region of a 5’UTR sequence affect protein expression. However, while this model performs well for the *fepB* dataset, it is unlikely to perform as well for other datasets where the sequence of the 5’UTR differs greatly from sequences on which it was trained. To access whether the features learnt by the model are transferable to other contexts/datasets, we explored the use of transfer learning.

We began by focusing on how easily the pre-trained *fepB* model could be fine-tuned for other sequence contexts (i.e. datasets of 5’UTRs whose sequences varied greatly from those in the *fepB* dataset). High-throughput genotype-to-phenotype experiments based on methods like Flow-seq often screen libraries of sequences with a constrained design (e.g. a large sequence containing a small variable region). In the Kuo dataset, the SD sequence within the RBS is a 9-nt variable region, flanked by different fixed sequences corresponding to different genetic contexts (i.e., *fepB*, *arti* and *dmsC*). Differences in the non-variable regions can have a strong influence on protein expression through different local mRNA interactions and folding (24,62,80,81) and become confounding effects during the training of a neural network. Although our first CNN-LSTM model was exclusively trained on the *fepB* context of the Kuo dataset, the features learnt might still be meaningful in different 5’UTR contexts, but some tuning of the network may be required.

**Figure 3.**
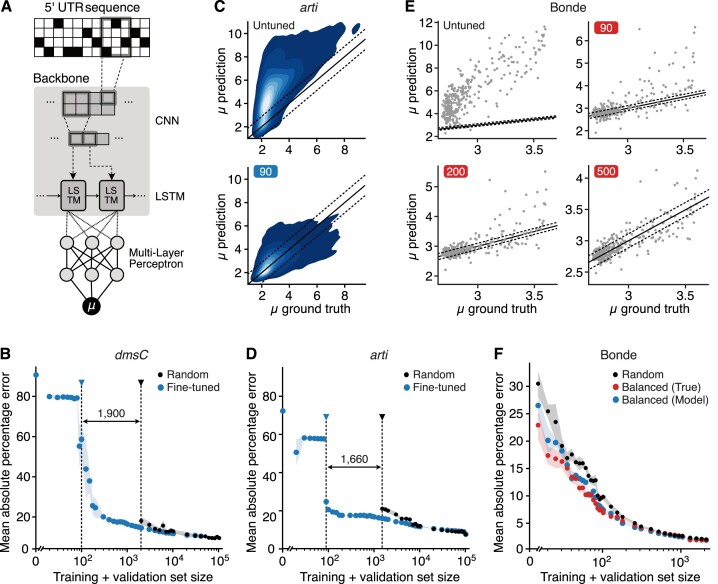
Adapting the deep learning model to new genetic and experimental contexts using transfer learning. (**A**) Architecture of the best performing neural network for predicting gene expression from 5’UTR sequence. Weights across all layers were adjusted during fine-tuning for different genetic and experimental contexts. (**B**) Test set (*dmsC* data) performance of the fine-tuned CNN-LSTM model (pre-trained on *fepB*)) versus a CNN-LSTM model trained from scratch (i.e., random initialization). Shaded areas denote the standard deviation observed when training three models using different random seeds. For the model trained from scratch, data is only shown when the predictions were not a constant value. Points at which constant values are not predicted of each model have highlighted by dotted lines. (**C**) Kernel density estimates of the test set (*arti* data) predictions of the mean log-fluorescence for the CNN-LSTM model trained on *fepB*), before fine-tuning (top) and after fine-tuning using 90 sequences (bottom). Predictions were averaged across three models trained using different random seeds. Solid black line shows *x* = *y* and the dashed black lines indicates the ±1.7 margin around it. The darkest shaded area containing 90% of the data. (**D**) Test set (*arti* data) performance of the fine-tuned CNN-LSTM model (pre-trained on *fepB*) versus a CNN-LSTM model trained from scratch (i.e. random initialization). Shaded areas denote the standard deviation observed when training three models using different random seeds. For the model trained from scratch, data is only shown when the predictions were not a constant value. Points at which constant values are not predicted of each model have highlighted by dotted lines. (**E**) Test set (Bonde data) predictions of the mean log-fluorescence for the fine-tuned CNN-LSTM model (trained on the entire Kuo dataset) fine-tuned using 0 (top left), 90 (top right), 200 (bottom left), 500 (bottom right) sequences. Predictions are on the *y*-axis, and ground truth is on the *x*-axis. Solid black line shows *x* = *y* and the dashed black lines indicates the ±1.7 margin around it. (**F**) Test set (Bonde data) performance of the CNN-LSTM model (pre-trained on the entire Kuo dataset) using different methods to select training sequences. Training sequences were selected either: (i) at random, (ii) using the known ground truth fluorescence values for each constructs to ensure our training set included sequences uniformly covering the full log-fluorescence range or (iii) using predictions from the pre-trained model before fine-tuning to ensure our training set included sequences uniformly covering the full predicted log-fluorescence range.

Transfer learning allows features learnt by a model to be adapted for use in a different context by adjusting neural network parameters in response to a limited number of samples from the new context (59). To see whether such an approach might work in our case, we investigated how efficient a simple fine-tuning procedure could be. We started with our pre-trained CNN-LSTM model for the *fepB* context (Figure 3A), and then tested it on other contexts after adjusting the weights using a few new training examples. To quantify the data requirements of this transfer leaning approach, we considered the *dmsC* and *arti* datasets from the Kuo experiment (Figure 1). For each of these, the data was collected in the same way as for the *fepB* dataset. The only difference being the fixed regions of the 5’UTR sequence. In addition to MAPE, we also used Spearman’s ρ coefficient when studying the efficiency of the fine-tuning protocol. Using Spearman’s ρ allowed us to compare the performance of models where the outputs had different units than our deep learning model (i.e., RBS Calculator v1, OSTIR and RBSeval).

Initially, the CNN-LSTM model performed poorly on the new contexts before fine-tuning, with a ρ = 0.73 and 0.62 for the *arti* and *dmsC* contexts, respectively, and a MAPE greater than 70% for both contexts (Figure 3B,D and Supplementary Tables S2 and S3). This demonstrates that the sequence motifs learned by the model during training on the *fepB* dataset are not directly generalisable to other contexts. This result may also stem from the fact that the CNN-LSTM model was trained on only the 5’UTR sequence alone. The N-terminus sequence of the expressed protein can affect translation initiation rate (23,24), but this information is unavailable to the model and so may hamper the accuracy that can be achieved.

This performance was still better than current RBS strength predictors such as RBSeval (ρ = 0.62 and 0.56 for the *arti* and *dmsC* contexts, respectively), OSTIR (ρ = 0.39 on both *arti* and *dmsC* contexts, respectively) and the RBS Calculator v1 (ρ = 0.14 and 0.48 for the *arti* and *dmsC* contexts, respectively) (Supplementary Tables S2 and S3), suggesting that these models are not as effective at predicting small RBS mutations. A possible explanation for this result is that the non-deep learning models rely on RNA secondary structure predictions, which might not adequately capture the effects of small mutations within the SD sequence. This hypothesis is supported by the poor correlation between protein levels and SD:aSD base pairing energy (ρ = −0.32 and −0.26 for the *arti* and *dmsC* contexts, respectively) and mRNA folding energy (ρ = 0.12 and 0.16 for the *arti* and *dmsC* contexts, respectively) (Supplementary Tables S2 and S3).

To assess the improvement that transfer learning can provide, the pre-trained CNN-LSTM model was fine-tuned alongside a model with the same architecture but random initial parameters (i.e. weights). As expected, the performance of the fine-tuned model improved with increasing numbers of new training examples (Figure 3B,D). However, the improvements were small after 90–200 fine-tuning examples, suggesting that the model had already been sufficiently refined for the new context (Figure 3C). This is highlighted by a drop in MAPE at this point of around 60% for the *dmsC* dataset and 40% for the *arti* dataset (Methods, Figure 3B,D). In contrast, at least 2000 training examples were required for the model trained from scratch to achieve a similar accuracy (Figure 3B,D). It is important to note that fine-tuning also regularizes training when data is limited (i.e. <2000 samples), as the neural networks trained from scratch often predict a constant value instead of learning meaningful patterns (e.g. see Supplementary Figures S4 and S5). Having such behaviour is undesirable as it hinders the use of commonly used techniques for improving model performance, like bagging, which involves averaging predictions from a group of independent models (82).

**Figure 4.**
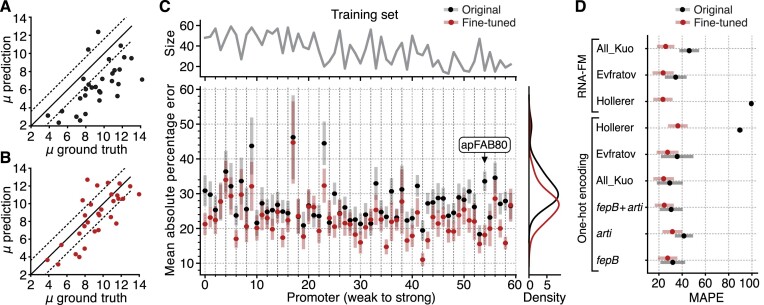
Transfer learning can effectively fine-tune our CNN-LSTM model for globally varying sequences with a small number of examples from new contexts. (**A**) Test set predictions of the mean log-fluorescence (Kosuri dataset, promoter apFAB80) using the CNN-LSTM model pre-trained on all Kuo data, before fine-tuning. (**B**) Test set predictions of the mean log-fluorescence (Kosuri dataset, promoter apFAB80) using the CNN-LSTM model pre-trained on all Kuo data, after fine-tuning using only 16 examples in the training set and 20 examples in the validation set. (**C**) Accuracy of the CNN-LSTM model pre-trained on all Kuo data, tested on each Kosuri promoter sub-dataset, before (black points) and after fine-tuning (red points). Top line graph shows the number of sequences in the training set for each promoter sub-dataset. Promoters are ordered (left to right) according to their strength (weak to strong). Error bars denote standard errors and were estimated using 500 bootstrap samples. Promoter apFAB80 has been highlighted. (**D**) Mean absolute percentage error (MAPE) for the entire Kosuri dataset before and after fine-tuning using every model studied. For embeddings, we used either an RNA Foundational Model (RNA-FM) or one-hot encoding of the 5’UTR sequence. Error bars denote the standard deviation for the MAPE across all promoters.

### Transfer learning for new sequence and experimental contexts

Transcription and translation are physically coupled in prokaryotes (83,84) allowing for regions of the promoter sequence downstream of the transcription start site to impact translational processes (5,80). As a result, the promoter sequence could become a confounding factor in high-throughput experiments where typically only a single promoter is used to drive transcription of a reporter gene.

To explore whether our fine-tuning approach could adapt to changes in promoter context, we made use of another dataset collected by Bonde and colleagues (64), where all mutations of a 6 nucleotide-long SD sequence in the RBS of the 5’UTR were characterised. Compared to the Kuo dataset on which our model was pre-trained, the Bonde dataset uses a different promoter (P_*proD*_ instead of P$_{{\it BBa\_J23100}}$), and has both a different fixed 5’UTR context around the RBS and different synonymous codon usage in the reporter protein (GFP) sequence. Furthermore, differences in the experimental setup (i.e. the FACS machine used to measure fluorescence) meant that the arbitrary fluorescence units used to report the protein expression strength also differed from the Kuo dataset (the Bonde test set took values in the interval [2.7, 3.6] arbitrary units).

We began by retraining our CNN-LSTM model using all data from the Kuo dataset (i.e. using all three 5’UTR contexts). This increased the model’s exposure to different genetic contexts around the RBS sequence. Fine-tuning this new model on the Bonde dataset revealed a large improvement in performance, with the MAPE decreasing initially from $95\% \pm 3\%$ to $9\% \pm 2\%$ after fine-tuning with a set of only 90 examples (Figure 3E,F). In contrast, the performance of the model trained from scratch always performed worse and often predicted only constant values (Supplementary Figure S6). The Bonde dataset lacked sufficient numbers of constructs to determine when the performance of these two models becomes comparable. This is consistent with a previous analysis of the Kuo dataset, which identified such a point between 10,000 and 20,000 sequences (Figure 3B,D). We found that incorrect predictions from the fine-tuned model initially tended to be large positive deviations from the ground truth. These differences were corrected as the number of fine-tuning examples increased (Figure 3E). These correspond to strong 5’UTRs, which are less frequent and therefore required more fine-tuning data in order to be observed by chance.

This result suggested that a fine-tuning dataset that includes constructs with a balanced range of fluorescence values would help accelerate the learning process. To test this, two strategies were used to select the training set. In the first, we used the known ground truth fluorescence values for each constructs and ensured our training set included sequences uniformly covering the range. In the second, we used predictions from the pre-trained model before fine-tuning as this placed no requirements on the new dataset to select sequences uniformly covering the full range of mean log-fluorescence. We found that both strategies consistently outperformed random sequence selection, with an average improvement in the MAPE of 31% when using the true fluorescence values and 20% using the model estimated values (Figure 3E,F). While access to experimental data might be limited, the ability to use a pre-trained model as a means to enhancing the training set for a new context is a low-cost and efficient way to accelerate the transfer learning process.

To understand the importance of the backbone module of the neural network (i.e., the CNN and LSTM layers), we conducted a variant of fine-tuning in which the backbone parameters for the CNN and LSTM layers were frozen and only the parameters of the MLP layers were updated. We hypothesized that the CNN-LSTM backbone was learning important sequence features, and the MLP capturing how those features should be combined and scaled. As many sequence features would be similar across contexts, a simpler fine-tuning of the MLP might allow for faster transfer learning as only the way that these existing learnt features are combined would need to be modified. We found this to not be the case, with this alternative fine-tuning procedure requiring at least 1,000 sequences to reduce the original error rate by 50% (Supplementary Figure S7). This highlights the importance of modifying parameters of the entire neural network and suggests that in addition to the conversion of fluorescent measurement units between datasets (i.e. scaling), which can be easily accommodated by modifications to the final MLP layer, new features are potentially learned during the fine-tuning process that require broader changes across the entire model.

### Transfer learning for diverse global 5’UTR contexts

So far, we have trained the CNN-LSTM model on datasets that include a small variable sequence within the 5’UTR. The lack of diversity across the full length of the 5’UTR could lead to issues in applying the model in broader sequence contexts. To address this, we examined whether the CNN-LSTM model was capable of adapting to 5’UTR sequences that differed across their full length (i.e. globally). We made use of a dataset collected by Kosuri *et al.* (5), which measured gene expression for a fully combinatorial library of 114 promoters and 111 RBSs. After extracting the mean log-fluorescence from the raw sequencing data and filtering out low quality sequences (Materials and methods), we were left with a collection of 60 promoters with varying numbers of associated RBSs. Each promoter sub-dataset was then divided into three sets: a training set (for fine-tuning, whose size varied from 12 to 61 sequences), a validation set (for fine-tuning early stopping, always consisting of 20 examples) and a test set (for evaluation, always consisting of 30 examples to minimize sampling variations).

We began by fine-tuning the CNN-LSTM model pre-trained on the entire Kuo dataset using data for each separate promoter from the Kosuri dataset. Despite the small amount of training data, the benefits of fine-tuning were still clear with a 15% decrease in MAPE for the apFAB80 promoter whose training set consisted of only 16 sequences (Figure 4A–C). Overall, for all promoters, fine-tuning decreased the MAPE of the predictions by 5% (*P* < 3.3 × 10^−6^, paired permutation testing of equal mean after and before fine-tuning) (Figure 4C). However, the improvements were not uniform across all promoters with some displaying poor accuracy even after fine-tuning. Furthermore, there was no clear relationship between promoter strength and tuning efficiency. Ignoring the issue of limited numbers of samples, this suggests that the features learnt during training on the full Kuo dataset were not sufficient to capture all of the biological phenomena present for every promoter in the Kosuri dataset. This is supported by the negative transfer observed for 12.3% of the promoters (Figure 4C), although the differences were not statistically significant (all *p* > 0.75 using paired permutation tests for equal mean after and before fine-tuning for each promoter) (Supplementary Figure S8).

To assess the amount of information learnt by the CNN-LSTM model, we compared its performance to a collection of additional models pre-trained on different datasets. First, to test the importance of highly diverse 5’UTR data for learning generalizable features, we compared the performance of the CNN-LSTM model trained on different combinations of 5’UTR contexts from the Kuo dataset. Models trained on a unique 5’UTR contexts (*fepB* or *arti*) had higher MAPE than models trained on combined 5’UTR contexts (*fepB* and *arti*, or All_Kuo). The model trained on all the 5’UTR contexts (All_Kuo) achieved the lowest MAPE of 24% across all promoter datasets (Figure 4C).

Increasing the sequence diversity of the dataset used for initial training means it is likely to encompass a wider range of biological factors/processes or sequence motifs that could influence gene expression. To test this, we conducted training and hyperparameter optimization for two additional CNN-LSTM models using two datasets where the 5’UTR composition was more substantially varied—the Evfratov (62) and Hollerer (54) datasets (Figure 1). This increase in sequence diversity during training did not lead to any significant improvement in model performance compared to datasets containing less sequence diversity (i.e., Kuo), with a higher MAPE for the Evfratov-trained model (27%, *P* = 1.2 × 10^−3^, paired permutation test for equal mean) and for the Hollerer-trained model (36%, *P* = 3.3 × 10^−6^, paired permutation test for equal mean). Remarkably, the limited fine-tuning examples were sufficient to adapt between the different experimental setups (i.e. fluorescence measurement units) used in the Hollerer dataset, which covered the interval [0, 1] capturing the area under the flipping ratio curve of a recombinase reporter system (54), and the Kosuri dataset, which covered the range [2, 15] arbitrary units measured using Flow-seq assay. Although such experiments could be improved by carefully controlling for data quality and quantity between datasets, our results suggest that that the biological phenomena observed when mutating the SD region and its local vicinity is sufficient for sequence models to extract useful generalizable patterns related to protein expression.

A further question we posed was whether fine-tuning could be improved by using additional biological information. To that end, we utilized embeddings computed by an RNA foundational model (FM) (70) instead of the raw 5’UTR one-hot encoding. The RNA-FM we used was trained through self-supervised learning using 23 million non-coding RNA sequences, which consisted of reconstructing for each RNA sequence the identity of a few (15%) masked nucleotides. The underlying transformer model makes use of the self-attention mechanism (85) to ensure the model learns contextual dependencies. Earlier work demonstrated the higher information content of these embeddings as input for CNNs on a large class of structure and function prediction tasks, including eukaryotic 5’UTR translational strength prediction (70). Furthermore, while the RNA-FM is not designed to predict interactions between separate RNAs like a transcript and the ribosome, it is important to recognize that secondary structures within a transcript (e.g. in the 5’UTR region) can impact the ability for other RNAs to interact (e.g. ribosomes). This feature is precisely what many synthetic RNA regulatory parts use to control translation initiation (e.g., toehold switches (86)). Therefore, the RNA-FM captures a useful feature that could impact RNA-RNA binding, as well as other factors implicated in overall protein expression strength (e.g. RNA degradation rate (87)).

We used their architecture and retrained the model on all the libraries considered so far, before fine-tuning it on the Kosuri dataset. Models trained using this embedding performed differently depending on the type of variation in the 5’UTR sequences. When trained on the Kuo dataset, where variation in sequences is limited to a 9 nt region, these embeddings resulted in a reduced ability to generalize with an increase of 27% in MAPE for the Kosuri dataset. Fine-tuning was also found to be slightly less efficient, with a significant increase in MAPE of 1.7% (*P* = 1.6 × 10^−5^, paired permutation test for equal mean) (Figure 4D). In contrast, using the RNA-FM embeddings for models pre-trained on globally varying 5’UTR sequences (i.e. Evfratov and Hollerer datasets) led to similar or better fine-tuning performance with a significant increase in mean accuracy of 13% after fine tuning for the Hollerer-trained model (*P* = 3.3 × 10^−6^ paired permutation test). This suggests such embeddings may be appropriate for sequences with a high degree of global diversity. Because the sequences from the Kuo dataset only vary by one-nucleotide, the embeddings computed by the RNA foundational model may not be suitable.

## Discussion

The ability to precisely control gene expression is crucial for the development of complex genetic circuitry that can reprogram living cells. Designing 5’UTR sequences is a popular approach to achieve this goal, but often requires the screening of many variants in order to find a sequence with precisely the desired expression strength. In this work, we have shown that deep learning models of 5’UTR strength can be derived from high-throughput experimental data and that they outperform many classical methods for predicting protein expression rate from 5’UTR sequence alone. Our analyses suggest that while current high-throughput assays provide sufficient information to accurately predict mean protein expression, higher order statistical information like the variance are not well captured. Furthermore, we have shown that the design of a Flow-seq experiment can bias the representations learned by a neural network, limiting the ability to use a trained model in different contexts. To solve this limitation, we demonstrated the potential of using transfer learning to adapt the neural network representations learnt for a large dataset to different contexts by adjusting the pre-trained neural network weights using a limited amount data from the new context. Although the performance of the fine-tuned model increases with the amount of the new data made available, we found that predictions can be greatly improved using as little as 36 new data points across different experimental conditions and setups. Our results suggest that a two-step approach, where a small-scale experiment is performed to fine-tune a pre-trained model in a new context, before it is then used to support sequence design.

We have previously illustrated the difficulty of inferring standard deviations from Flow-seq datasets (57), which is due to higher-order moments being more sensitive to small deviations in the distributions from which they are inferred. Because the Flow-seq methodology requires a coarse-grained discretization of cell fluorescence into a small number of bins (usually between 6–12), a limited number of cells being sorted into these bins, and a relatively small number of sequencing reads recovered for each genetic variant, there is a large amount of noise in the raw distributions from the Flow-seq data. This is currently unavoidable, and while average fluorescence values are robust to this noise, it does causes issues for higher order moments. To effectively learn standard deviations, more accurate measurements of this feature are required. These could be obtained by performing flow cytometry experiments for each genetic variant separately, to capture accurate fluorescence distributions. Unfortunately, this would not be feasible for the size of the libraries we consider here. Alternatively, a more fine-grained binning and deeper sequencing could be performed per library to reduce the impact of the discretisation of the Flow-seq methodology. Again, such experiments are financially prohibitive at present, but may in the future become feasible.

The transfer learning protocol used here employs a lowered learning rate. While this approach is appealing due to its simplicity, it could be refined by using more advanced methods from few-shot learning to decrease the amount of training data needed from the new context (88). The transfer learning step itself could also suggest the most informative sequences to characterize, using for example different acquisition functions to reduce epistemic uncertainty (89). Furthermore, the deep learning models we used only considered the 5’UTR sequence alone, ignoring surrounding sequence information (e.g., the downstream protein coding region). While it is remarkable that only a handful of sequences are capable of adapting such models to new contexts, integrating information from the surrounding sequence could help increase model generalization.

While improvements to the transfer learning approach may be helpful, fundamentally the underlying deep neural network model still needs access to a large experimental dataset for initial training. An interesting future direction to overcome this requirement would be to blend the capabilities of mechanistic and deep learning models to reduce the experimental data needed for initial training and transfer learning. Specifically, a mechanistic model like OSTIR could be used to generate large synthetic datasets for new organisms or genetic contexts that can then be used to ‘prime’ the deep learning model such that smaller quantities of experimental data are then needed to reach an acceptable performance level. The use of synthetic data and mechanistic simulations has been shown to be extremely useful in other areas of deep learning (e.g., robotics (90)), and this hybrid approach may offer the means to reduce the experimental cost of developing deep learning models for biological systems.

It is known that the N-terminus sequence of a protein can affect translation initiation rate (23,24). Here, we purposefully focused on the 5’UTR alone, to better understand how general sequence features of this region can be used to predict protein translation rate irrespective of the protein being expressed. However, this will hamper the ability for our deep learning model to capture interactions between the 5’UTR and protein coding region and may account for some of the differences we observed in our model predictions across sequence contexts. Expanding this work to include a wide array of experimental data covering diverse N-terminus sequences would be valuable for elucidating the importance of these interactions and assessing whether this information can help improve generalization of the model further.

Another interesting extension of this work would be to consider broader biological and experimental contexts (e.g., see Figure 1). Our focus here has been on examples where large-scale dataset have been available, allowing us to explore changes in local sequence context, host strain and the experimental machinery used to measure fluorescence. The growing realization of the influence of plasmid backbone (42), the indirect impact of other proteins being expressed that cause metabolic burden (91), and compositional effects at the level of a genetic circuit containing many interacting parts (4) remains to be explored.

A fundamental issue when using large neural network models for biological design is the lack of mechanistic insight they provide. This is beginning to be addressed with the emergence of explainable AI (xAI) where the internal structure of a trained model is analysed to understand the features learnt, their interactions, and their importance when making predictions (92). In relation to this work, approaches focused on feature attribution (93) and propagation-based methods (94) that allow for relationships to be inferred between sequence motifs would be relevant for inferring possible mechanistic roles of sub-sequences (95). However, challenges remain in how best to apply these new methods and it is not uncommon for nonsensical results to emerge. Even so, modifying the architecture of our deep neural network to better accommodate interpretability (96) or applying xAI methods to an existing model, would be an interesting future direction to assess how features critical for accurate prediction of 5’UTR strength shift across contexts and the underlying biological mechanisms that might explain these changes.

In summary, data-centric approaches to biological design are revolutionizing how we design experiments (97,98), proteins (99,100) and genetic parts/circuits (44,51). However, being able to reuse and re-purpose machine learning models will be vital for broadening their reach beyond the constrained experiments used for training. The work presented here offers a practical approach to overcoming this issue and will support the development of ‘context-aware’ deep learning models tailored to the context in which they are being used to accelerate genetic circuit design workflows and reduce the need for trial-and-error when scaling the complexity of our biological designs.

## Supplementary Material

gkae491_Supplemental_File

## Data Availability

All data associated with this study has been uploaded as three zip files to Zenodo (DOI: 10.5281/zenodo.11081336). The ‘rebeca.zip’ file contains a snapshot of the rebeca package which can be used to train, fine tune and test the CNN-LSTM model used in this study. The ‘datasets.zip’ file contains the compiled sequence to expression datasets from across all Flow-seq expressions considered in this study. Finally, the ‘analysis.zip’ file contains all data files and jupyter notebooks necessary to reproduce our analysis. Each Flow-seq study has a dedicated folder (e.g., ‘fepB’) with two sub-folders: 1. The ‘data_split’ folder, which contains the steps necessary to split the Flow-seq data for our ML experiments (a ‘readme.txt’ file describes the input and output files and a jupyter notebook is available to reproduce the data split); 2. The ‘data_analysis’ folder, which contains a jupyter notebook and the necessary input files to reproduce the analysis of our experiments. The development code repository of the rebeca package is available at: https://gitlab.com/Pierre-Aurelien/rebeca.
