# Supplementary material for: Transfer learning for cross-context prediction of protein expression from 5’UTR sequence
Source: Nucleic Acids Res. 2024 Jun 12;52(13):e58. doi: 10.1093/nar/gkae491 (PMC11260469; doi:10.1093/nar/gkae491)
Supplement: gkae491_Supplemental_File [file gkae491_supplemental_file.pdf]

Transfer learning for cross-context prediction of protein expression from 5'UTR sequence

Pierre-Aurélien Gilliot[1] **and Thomas E. Gorochowski**[1,2]

[1]**School of Biological Sciences, University of Bristol, 24 Tyndall Avenue, Bristol, BS8 1TQ, UK**

[2]**BrisEngBio, School of Chemistry, University of Bristol, Cantock's Close, Bristol, BS8 1TS, UK**

CONTENTS

Supplementary Notes **2**

Supplementary Note 1: Predicting the standard deviation of protein expression . . . 2

Supplementary Tables **4**

Supplementary Table 1: Hyperparameter optimisation results for the *fepB* context . . . 4

Supplementary Table 2: Performance metrics for the *arti* context . . . 5

Supplementary Table 3: Performance metrics for the *dmsC* context . . . 6

Supplementary Figures **7**

Supplementary Figure 1: Accounting for the uncertainty of the neural network random initialisation . . . 7

Supplementary Figure 2: Precision of Flow-seq estimates . . . 8

Supplementary Figure 3: Predictions on *fepB* activity cliffs using the RBS calculator v2.1 . . . 9

Supplementary Figure 4: Visualising *dmsC* test set predictions . . . 10

Supplementary Figure 5: Visualising *arti* test set predictions . . . 11

Supplementary Figure 6: Visualising test predictions for the Bonde dataset . . . 12

Supplementary Figure 7: Comparing fine-tuning procedures on the Bonde dataset . . . 13

Supplementary Figure 8: Negative transfer on the Kosuri dataset . . . 14

References **15**

Supplementary Note 1: Predicting the standard deviation of protein expression

The Flow-seq experiments used to generate the Kuo dataset involved sorting genetic variants into several contiguous bins covering varying levels of output fluorescence. This allows for higher-order statistical information to be measured such as the standard deviation of the fluorescence distribution by analysing the spread of genetic variants across the bins. Such information is indicative of the level of variability or noise across the population, and is potentially useful for developing parts to control variation in gene expression.

We explored whether it was possible for our deep learning model to learn the standard deviation of the fluorescence output from 5'UTR seqeunce. To assess whether our model could be used to predict this feature, the original CNN-LSTM model was modified to include an additional neuron in the output layer to predict the standard deviation of the log-fluorescence distribution. This new head enables the model to learn a distribution by minimizing the distance to the ground truth distribution. This distance was measured using the Kullback Leibler (KL) divergence [1]. After optimizing the hyperparameters of this new architecture, we assessed the predictions of two instances of this model: 1. trained using the original dataset, and 2. trained using an altered version of this dataset where the standard deviation information was shuffled randomly between samples while the mean fluorescence values were maintained (allowing comparison against a randomised baseline during training).

For both models, the accuracy when predicting the mean log-fluorescence was similar with a difference in MAPE of only 0.03%, which was not statistically significant ($p > 0.7$, Welsh t-test) **(Figure 2F)**. When predicting the standard deviation, the model trained using the shuffled dataset performed slightly worse, with a MAPE = 48.9% versus 46.3% for the normal dataset, respectively. However, this difference of 2.6% is still small in comparison to the typical uncertainty in the fluorescence standard deviation predictions, which was calculated to be approximately 52%. This uncertainty was estimated the same way as for the mean log-fluorescence, by computing the 99.7% confidence intervals for the MAPE of the log-fluorescence standard deviation (**Supplementary Figure 2**). The similarity between errors suggests that either the model is not well-suited to learning the standard deviation, or that the experimental data does not contain sufficient information for estimating this feature. Indeed, had the data contained a learnable signal, the performance of the model trained on the original data would have been higher than the model trained on shuffled data. Additionally, the model trained on the original data was found to perform worse than a simple baseline model, which always returns the average standard deviation of the log-fluorescence with a Mean Absolute Error (MAE) $= 0.210 \pm 0.001$ (**Figure 2C**).

It is unlikely that the CNN-LSTM model is unable to capture core features of the data, as we allowed for the neural network to expand its capacity during the hyperparameter optimization. Instead, it is more likely that this issue is due to unavoidable errors in the estimation of the standard deviations from the Flow-seq

data. These stem from the discrete nature of the approach (i.e., sorting of cells into a small number of bins corresponding to fixed ranges in fluorescence) and the often low numbers of reads obtained for some genetic constructs [2, 3].

Hyperparameter	Search Space	
	CNN-LSTM	CNN
CNN Channels	[64, 128, **256**, 512]	[64, 128, **256**, 512]
CNN Layers	[1, **2**, 3]	[1, 2, **3**]
CNN Kernel	[4, 6, **8**, 10]	[4, 6, 8, **10**]
CNN Pool	[**1**, 2]	[**1**, 2]
LSTM Hidden Size	[5, 10, 25, **50**, 100, 200, 500]	∅
BILSTM	[**True**, False]	∅
Activation	[ReLU, ELU, **LeakyReLU**]	[ReLU, ELU, **LeakyReLU**]
MLP Hidden Size	[64, **128**, 256, 512, 1024]	[64, 128, 256, 512, **1024**]
MLP Layers	[**1**, 2, 3]	[1, **2** ,3]
Dropout Rate	[0..**0.28**..0.5]	[0..**0.36**..0.5]
Learning Rate	[1e-4..**3e-4**..1e2]	[1e-4..**2.6e-4**..1e2]
Batch Size	[**32**, 64]	[32, **64**]
Number of parameters	1, 180, 837	8, 659, 713

Supplementary Table 1: Hyperparameter optimisation results for the *fepB* context (optimal parameters in bold).

Algorithm	Metrics		
	MAPE	Mutual Information	Spearman's ρ
RBSeval	—	1.16 ± 0.02	0.616 ± 0.005
OSTIR	—	0.98 ± 0.02	0.389 ± 0.005
CNN-LSTM (*fepB* trained, before fine-tuning)	0.720 ± 0.001	$\mathbf{1.30 \pm 0.02}$	$\mathbf{0.727 \pm 0.003}$
ΔG SD:aSD base pairing	—	0.43 ± 0.01	-0.317 ± 0.007
ΔG mRNA folding	—	0.57 ± 0.01	0.122 ± 0.007
RBS Calculator v1	—	0.33 ± 0.01	0.143 ± 0.006

Supplementary Table 2: Performance metrics for the *arti* context.

Algorithm	Metrics		
	MAPE	Mutual Information	Spearman's ρ
RBSeval	—	1.05 ± 0.02	0.560 ± 0.005
OSTIR	—	1.01 ± 0.02	0.391 ± 0.006
CNN-LSTM (*fepB* trained, before fine-tuning)	0.904 ± 0.004	$\mathbf{1.14 \pm 0.02}$	$\mathbf{0.619 \pm 0.005}$
ΔG SD:aSD base pairing	—	0.35 ± 0.01	-0.257 ± 0.007
ΔG mRNA folding	—	0.48 ± 0.01	0.156 ± 0.006
RBS Calculator v1	—	1.05 ± 0.02	0.486 ± 0.005

Supplementary Table 3: Performance metrics for the *dmsC* context.

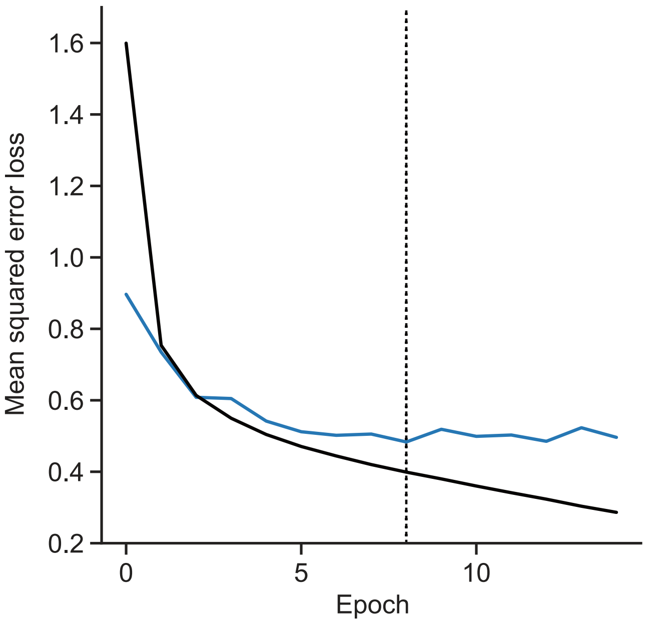

Supplementary Figure 1: Learning Curves. Mean squared error loss for the validation dataset (blue curve) and training dataset (black curve) during model training of the best CNN-LSTM model when predicting the log-fluorescence mean. Dashed line indicates the epoch chosen to stop training using our early stopping criteria (**Methods**).

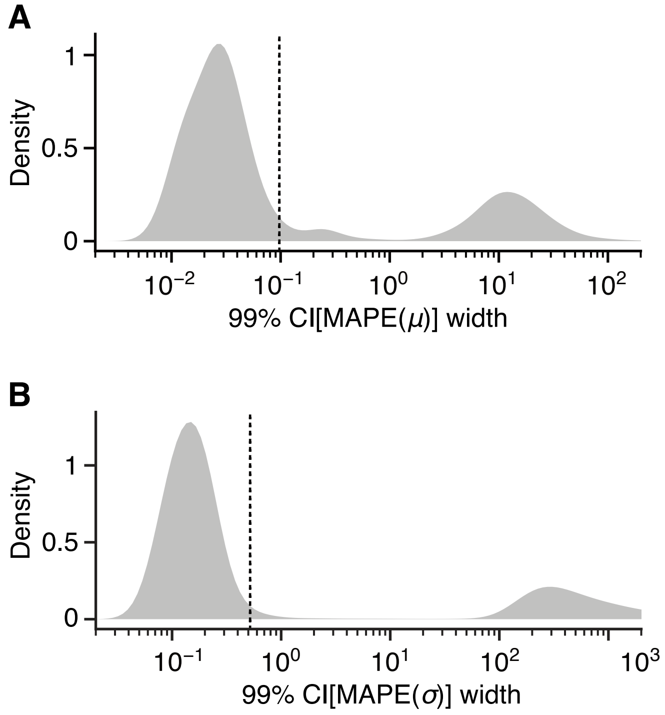

Supplementary Figure 2: Precision of Flow-seq estimates. (**A**) Distribution of the standard error width when computing the mean percentage error (MAPE) associated with the maximum likelihood estimates of the fluorescence mean (μ) for each sequence from the *fepB* context of the Kuo experiment. (**B**) Distribution of the standard error width when computing the MAPE associated with the maximum likelihood estimates of the standard deviation (σ) for each sequence from the *fepB* context of the Kuo experiment. Dashed line indicates the median 99.7% confidence interval for each statistic: 0.097 for the log-fluorescence mean and 0.520 for the log-fluorescence standard deviation.

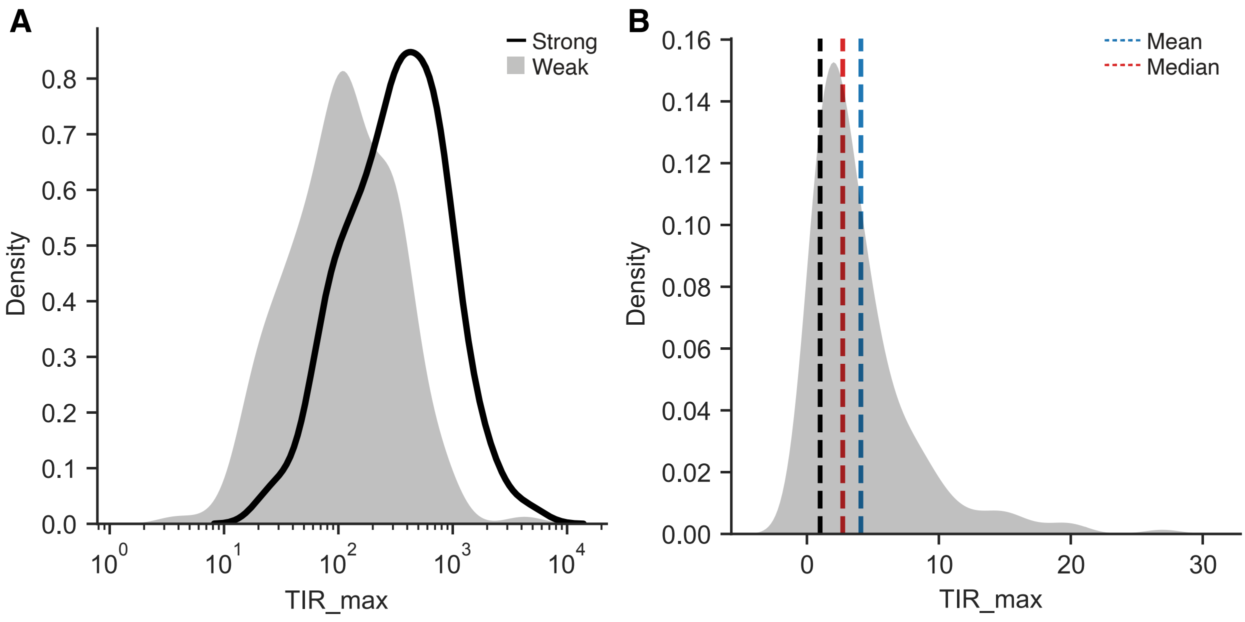

Supplementary Figure 3: Predictions on activity cliffs using RBS calculator v2.1. (**A**) Distributions of maximum translation initiation rates (TIR_max) using the RBS calculator v2.1 for the weak (mean log fluorescence $\mu < 3$) and strong (mean log-fluorescence $\mu > 6$) 5'UTR sequences from the *fepB* test set. (**B**) Distribution of the TIR_max ratio between pairs of activity cliffs from the *fepB* test set. The ratio was taken between the TIR_max of the strong 5'UTR and the TIR_max of the weak 5'UTR sequence. Both sequences only differ by only one mutation. Mean (blue dashed line) = 4, median (red dashed line) = 2.7, black solid line = 1.

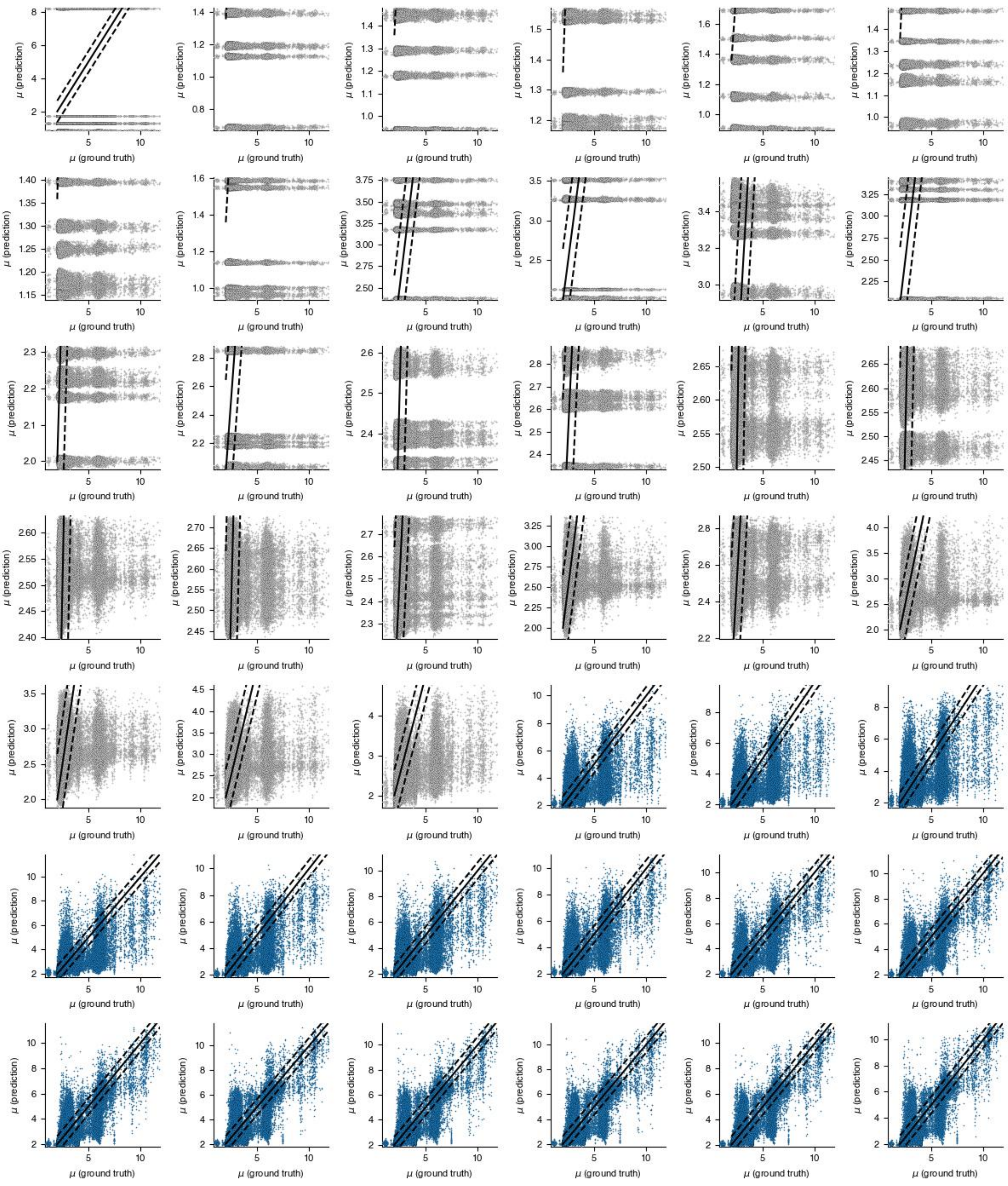

Supplementary Figure 4: Visualising *dmsC* test set predictions. Prediction of the hybrid CNN-LSTM neural network trained from scratch on the *dmsC* dataset with varying numbers of examples in the training and validation set: From left to right, top to bottom: 10, 20, 30, 40, 50, 60, 70, 80, 90, 100, 125, 150, 175, 200, 300, 400 ,500, 600, 700, 800, 900, 1,000, 1,250, 1,500, 1,750, 2,000, 3,000, 4,000, 5,000, 6,000, 7,000, 8,000, 9,000, 10,000, 20,000, 30,000, 40,000, 50,000, 60,000, 70,000, 80,000, and 90,000 . Predictions where the range matches the input fluorescence range are colored in blue. Solid black line shows $x = y$ and dashed line indicate the margin of tolerance to place a construct into one of four categories (**Methods**).

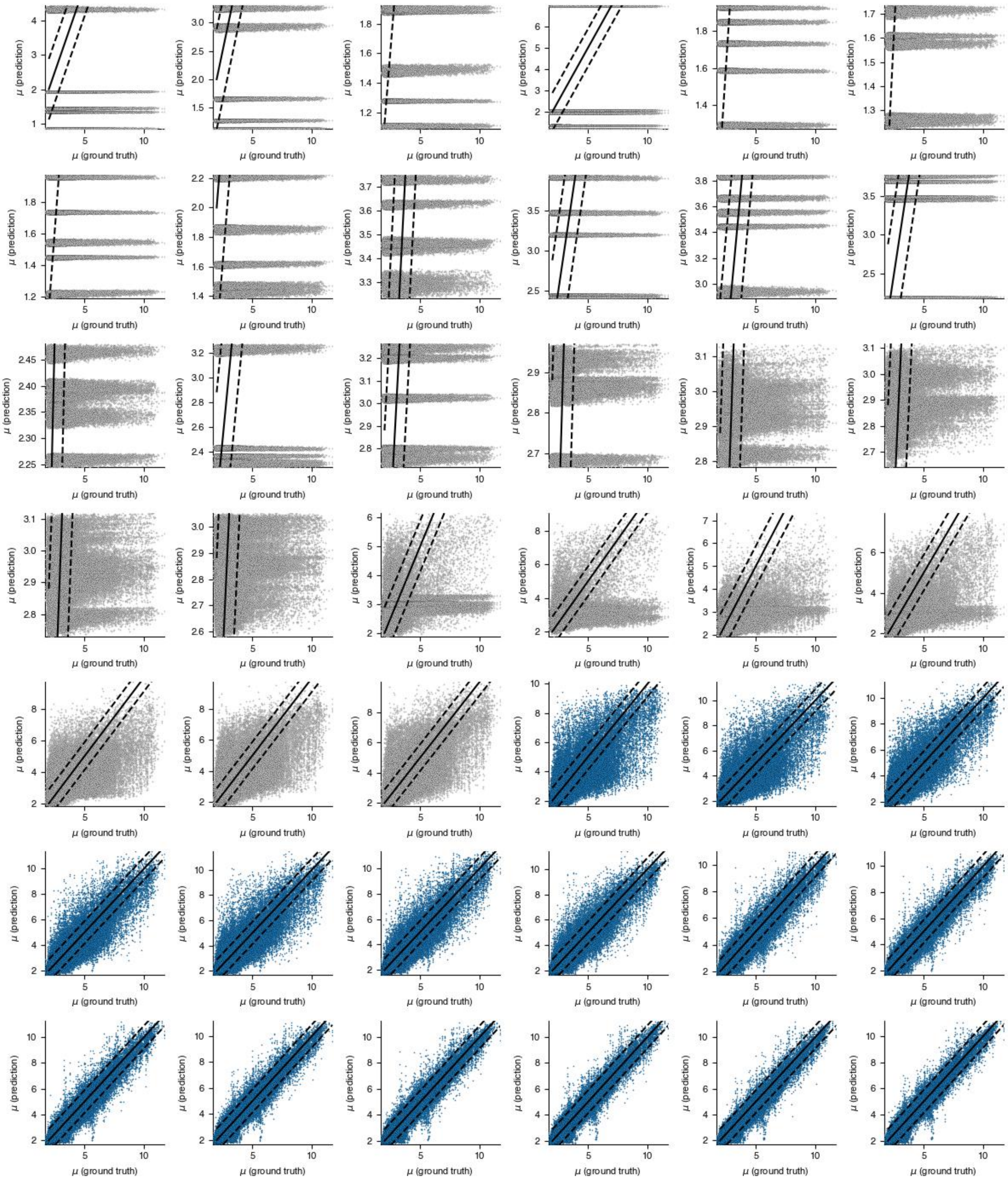

Supplementary Figure 5: Visualising *arti* test set predictions. Prediction of the hybrid CNN-LSTM neural network trained from scratch on the *arti* dataset with varying numbers of examples in the training and validation set: From left to right, top to bottom: 10, 20, 30, 40, 50, 60, 70, 80, 90, 100, 125, 150, 175, 200, 300, 400, 500, 600, 700, 800, 900, 1,000, 1,250, 1,500, 1,750, 2,000, 3,000, 4,000, 5,000, 6,000, 7,000, 8,000, 9,000, 10,000, 20,000, 30,000, 40,000, 50,000, 60,000, 70,000, 80,000, and 90,000. Predictions where the range matches the input fluorescence range are colored in blue. Solid black line shows $x = y$ and dashed line indicate the margin of tolerance to place a construct into one of four categories (**Methods**).

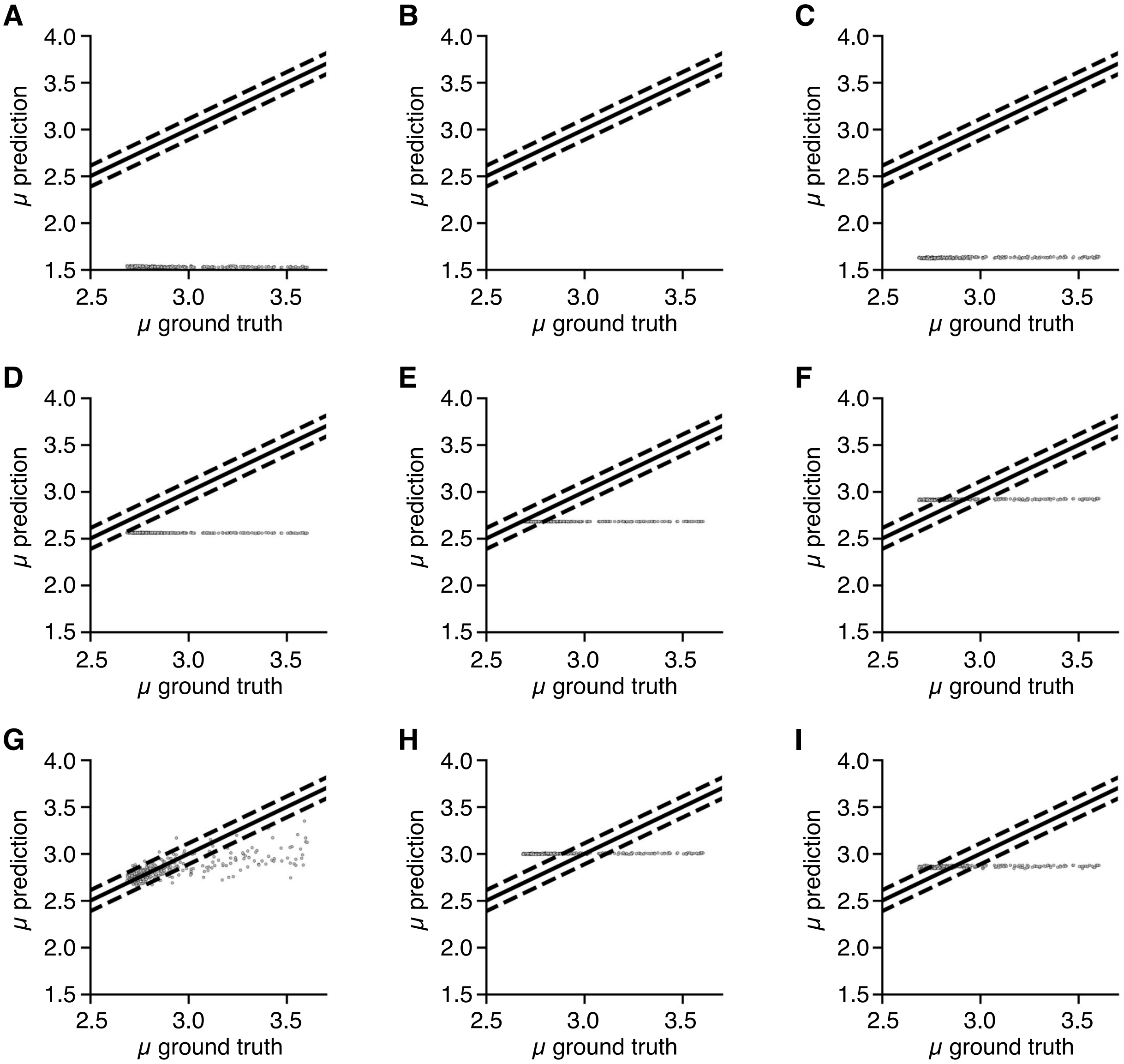

Supplementary Figure 6: Visualising test predictions for the Bonde dataset. Prediction of the hybrid CNN-LSTM neural network trained from scratch on the Bonde dataset with varying amounts of examples in the training and validation set: (**A**) 50, (**B**) 60, (**C**) 70, (**D**) 80, (**E**) 90, (**F**) 200, (**G**) 500, (**H**) 1,000, and (**I**) 2,000. Solid black line shows $x = y$ and dashed line indicate the margin of tolerance to place a construct into one of four categories (**Methods**).

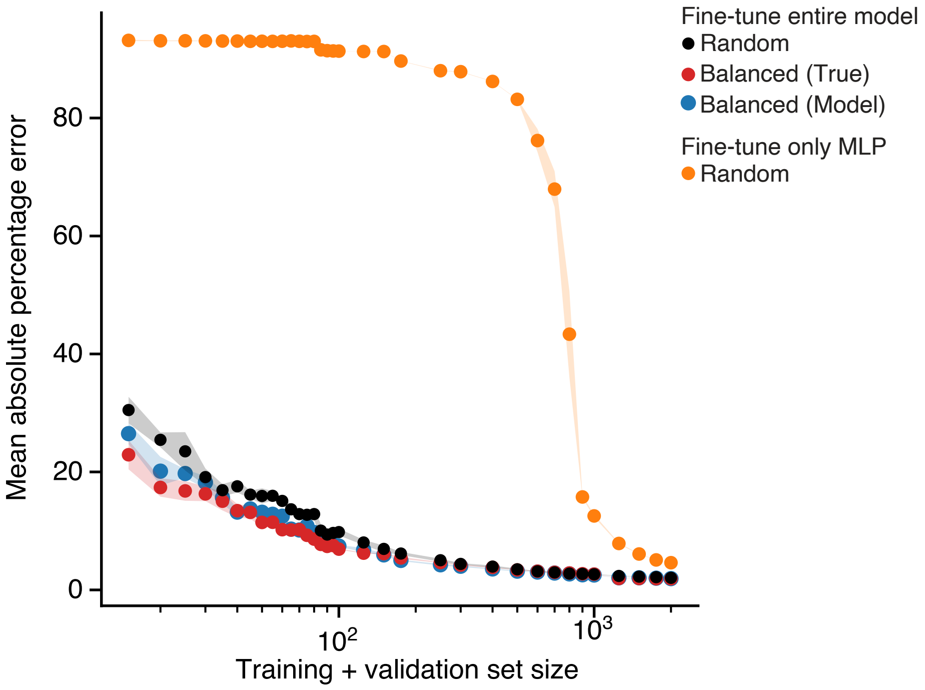

Supplementary Figure 7: Comparing fine-tuning procedures on Bonde dataset. Performance on the Bonde dataset of the hybrid CNN-LSTM pre-trained on the entire Kuo dataset and fine-tuned by either updating all model parameters or only the parameters of the last multi-layer perceptron (MLP). Different options for selecting sequences for fine-tuning were explored. Sequences were either selected at random, or by selecting sequences with a balanced range of fluorescence using the true values from the ground truth data, or via prediction using the CNN-LSTM model before fine-tuning. Fine-tuning of the MLP layer alone sees a sharp increase in accuracy after approximately 800 fine-tuning examples. It should be noted that this constitutes approximately 20% of the entire dataset for the new Bonde sequence context. Therefore, this transition may correspond to the MLP itself having learnt sufficient features to make accurate predictions, rather than the exploitation of features encoded in the fixed CNN-LSTM backbone.

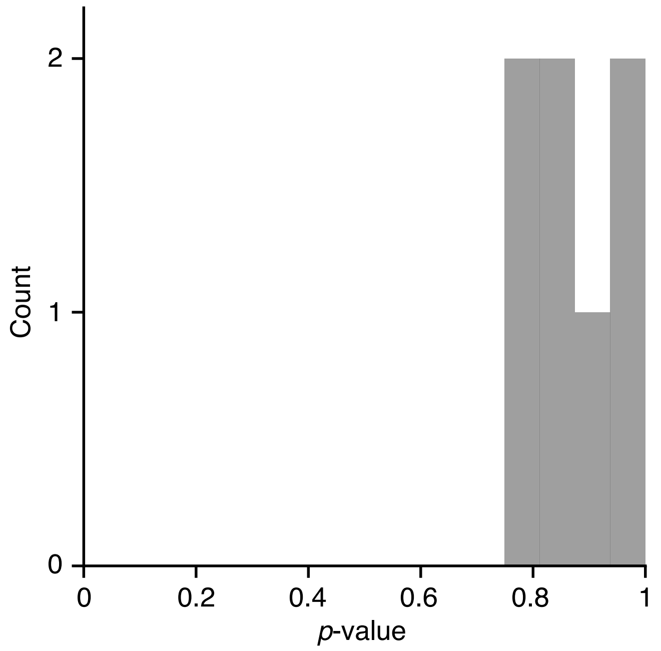

Supplementary Figure 8: Negative transfer on Kosuri dataset. Distribution of *p*-values using paired permutation tests for equal mean before and after fine-tuning for promoter 0 to promoter 7 from the Kosuri dataset. The predictions are taken from the CNN-LSTM model trained on the entire Kuo dataset.

REFERENCES

[1] Kingma, D. P. and Welling, M. (2013) Auto-Encoding Variational Bayes. *arXiv:1312.6114,*.

[2] Höllerer, S., Papaxanthos, L., Gumpinger, A. C., Fischer, K., Beisel, C., Borgwardt, K., Benenson, Y., and Jeschek, M. (2020) Large-Scale DNA-based Phenotypic Recording and Deep Learning Enable Highly Accurate Sequence-Function Mapping. *Nature Communications,* **11**(1), 3551.

[3] Gilliot, P.-A. and Gorochowski, T. E. (2023) Effective design and inference for cell sorting and sequencing based massively parallel reporter assays. *Bioinformatics,* **39**(5), btad277.
